# Role of Point-of-Care Testing in Reducing Time to Treatment Decision-Making in Urgency Patients: A Randomized Controlled Trial

**DOI:** 10.5811/westjem.2019.10.43655

**Published:** 2020-02-25

**Authors:** Wansiri Chaisirin, Preechaya Wongkrajang, Tenzin Thoesam, Nattakarn Praphruetkit, Tanyaporn Nakornchai, Sattha Riyapan, Onlak Ruangsomboon, Sathima Laiwejpithaya, Kavisara Rattanathummawat, Rungrudee Pavichai, Tipa Chakorn

**Affiliations:** * Siriraj Hospital, Mahidol University, Department of Emergency Medicine, Bangkok, Thailand; † Siriraj Hospital, Mahidol University, Department of Clinical Pathology, Bangkok, Thailand; ‡ Siriraj Hospital, Outpatient unit, Bangkok, Thailand

## Abstract

**Introduction:**

Shortening emergency department (ED) visit time can reduce ED crowding, morbidity and mortality, and improve patient satisfaction. Point-of-care testing (POCT) has the potential to decrease laboratory turnaround time, possibly leading to shorter time to decision-making and ED length of stay (LOS). We aimed to determine whether the implementation of POCT could reduce time to decision-making and ED LOS.

**Methods:**

We conducted a randomized control trial at the Urgency Room of Siriraj Hospital in Bangkok, Thailand. Patients triaged as level 3 or 4 were randomized to either the POCT or central laboratory testing (CLT) group. Primary outcomes were time to decision-making and ED LOS, which we compared using Mann-Whitney-Wilcoxon test.

**Results:**

We enrolled a total of 248 patients: 124 in the POCT and 124 in the CLT group. The median time from arrival to decision was significantly shorter in the POCT group (106.5 minutes (interquartile [IQR] 78.3–140) vs 204.5 minutes (IQR 165–244), p <0.001). The median ED LOS of the POCT group was also shorter (240 minutes (IQR 161.3–410) vs 395.5 minutes (IQR 278.5–641.3), p <0.001).

**Conclusion:**

Using a point-of-care testing system could decrease time to decision-making and ED LOS, which could in turn reduce ED crowding.

## INTRODUCTION

Emergency department (ED) crowding has become a major worldwide issue. Many previous studies have shown that ED crowding resulted in delayed management, thereby affecting overall healthcare quality.^1^ Examples of the effect of ED crowding are delayed time to antibiotics administration in patients with pneumonia^2^ and increased adverse cardiovascular outcomes in patients with chest pain.^3^ Such delays may lead to higher morbidity and mortality among emergency patients.^4^ One way to solve this is to improve patient flow by minimizing ED length of stay (LOS). Shorter LOS is associated with higher patient satisfaction^5^ and a decrease in mortality and morbidity.^6^,^7^

Laboratory turnaround time (TAT) is defined as the time from blood sample accessing to reporting of results.^8^ Prolonged TAT may cause delayed treatment and increased LOS, ultimately leading to ED crowding. Point-of-care testing (POCT), which can be performed immediately at bedside, can shorten TAT and LOS.^9^ Several studies have reported that the median (interquartile range [IQR]) TAT of POCT was shorter than that of the central laboratory test (CLT).^10^–^14^ One study also found that POCT could decrease mean and median LOS.^15^ However, many studies have found no significant difference in LOS between patients using POCT and CLT.^16^,^17^

Due to the contrasting results of those previous studies, our goal was to evaluate the effect of POCT using the i-STAT system (Abbott Laboratories, Abbott Park, IL) on time to decision-making and LOS in urgency patients.

## METHODS

### Study population

This randomized controlled study was conducted at the urgency room of Siriraj Hospital by the Department of Emergency Medicine and Clinical Pathology of the Faculty of Medicine, Siriraj Hospital. The hospital is the largest tertiary-care university hospital in Bangkok, Thailand, accommodating over 2,800,000 outpatient visits and around 18,000 ED visits per year. We included patients if they were (1) over 18 years old, (2) classified as triage level 3 (urgency) and 4 (semi-urgency) by the Siriraj Adult Triage System (Table 1), and (3) clinically required electrolyte blood tests (sodium, potassium, chloride, bicarbonate). We excluded pregnant, traumatic and bedridden patients.

### Sample size calculation

Per a previous study by Loten et al,^18^ turnaround time of central lab testing was assumed to be about 1.5 hours. To detect a time difference between two groups of approximately 30 minutes, with *p* = 0.05, power of 80% and 1:1 randomization, 104 participants per group was required. After adding another 20% to prevent missing data, the estimated sample size per group was 124.

**Population Health Research Capsule**

What do we already know about this issue?

The implementation of point-of-care testing (POCT) could provide a decrease in laboratory turnaround time compared to central lab testing.

What was the research question?

To evaluate the effect of POCT on time to decision-making (TOD) and emergency department length of stay (ED LOS) in urgency patients.

What was the major finding of the study?

This study demonstrated a significant decrease in the lab turnaround times, time to decision, and ED-LOS after the implementation of POCT.

How does this improve population health?

Using POCT could result in better utilization of resources, more patient access, and potentially less ED crowding.

### Outcomes

The primary outcomes were time to decision-making (TOD) and ED LOS. TOD is the period from ED arrival to the time the physician made a decision on patient treatment and recorded it in the physician order sheets. We defined LOS as the period from ED arrival to the time that the patient left the ED. The secondary outcomes were satisfaction of physicians, nurses, and patients, assessed by a questionnaire. The satisfaction scale was graded from 1 (very poor) to 5 (excellent). (See supplementary appendix.).^19^ A project investigator would assess the satisfaction scale from the physician, the nurse, and the patient after all treatment was completed and before the patient was discharged.

### Study Flow

At the urgency room of Siriraj Hospital, patients triaged level 3 and 4 are assessed by attending physicians who determine whether the patients require any lab tests. Once blood electrolyte was ordered, the nursing staff would allocate these patients and notify the project researchers for patient recruitment. We then obtained written informed consent from eligible patients or their relatives. Included patients were randomized to either the CLT group or the POCT group in a 1:1 ratio. Randomization was generated by software in blocks of four using sealed opaque envelopes. Both groups received standard therapy for any medical problem.

#### Central lab test (CLT) group

In this group, blood samples were drawn and transferred to the central lab as usual. The nursing staffs would report the results to the attending physician once the results were reported online.

#### POCT system group

Patients in this group also had their blood drawn by nurses. The blood samples were then analyzed using the POCT system in the Urgency Room. Printed results were then attached to the patient’s medical record. The nursing staff would report the results to the attending physicians as soon as possible. If other laboratory profiles were ordered, the blood samples were also sent to the central lab for those results.

For this study we used the i-STAT system (Abbott Laboratories, Abbott Park, IL), a portable blood analyzer composed of a handheld device and cartridges. A test is done by inserting 2–3 drops of blood into the cartridge; the cartridge is then inserted into the handheld device. The results can usually be read within five minutes for most cartridges. The device operates with single-use, disposable test cartridges. The CHEM 8+ cartridge used in this study consisted of sodium, potassium, chloride, ionized calcium, total CO_2_, glucose, blood urea nitrogen (BUN), and creatinine. The precision and accuracy of the tests in determining sodium, potassium, and BUN were found to be acceptable.^12^,^14^ Likewise, the POCT analyzer used in our study had been verified and validated to be precise and accurate compared to the hospital’s central lab analyzer prior to the commencement of this study.

The nurses were trained to operate the POCT system prior to the study. And quality control was assessed as per the manufacturer’s guidelines before trial initiation and during the data collection period by an Abbott representative. The POCT handhelds and cartridges were supported by Transmedic Thailand Co, Ltd. For both groups, the attending physicians would make the decisions on patients’ management according to the lab results. Project researchers collected the data required and interviewed the physicians, nurses, and the patients for their feedback and level of satisfaction.

### Data collection

We recorded baseline characteristics. Also recorded were the times of ED arrival, initial assessment by attending physicians, and first blood draw. We also recorded the following times: lab results were reported; when the physician was notified; and the time of decision-making.

### Statistical analysis

**Figure 1:**
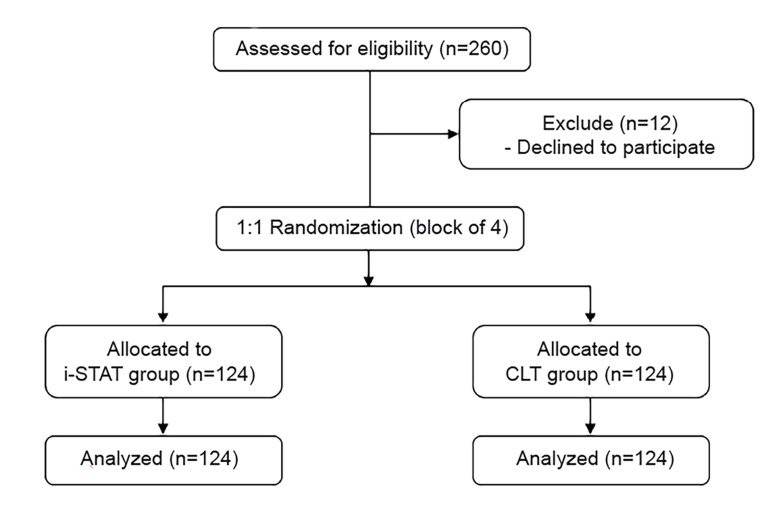
Study flow.

We performed all analyses on an intention-to-treat basis. We present a flow diagram of progress through the phases of the trial, as suggested by the CONSORT 2010 statement (Figure 1). Demographics and baseline characteristics of all randomized participants were summarized by treatment arms. Continuous variables were presented as mean and standard deviation. We described categorical variables as frequencies and percentages, while time intervals were presented as median and interquartile ranges (IQR). We compared intervals between the two groups using the Mann-Whitney-Wilcoxon test, while Pearson chi-square test was used to compare qualitative variables.

All statistical tests were performed using PASW 18.0 statistics for windows (SPSS Inc., Chicago, IL). P-value of less than 0.05 was considered of statistical significance.

This research was reviewed by the Thai Clinical Trials Registry (TCTR) Committee. TCTR identification number is TCTR20170324005 (prospectively registered on March 24, 2017). Ethics approval for the study and a research approval code, 802/2559 (EC4), were provided by the Siriraj Institutional Review Board.

## RESULTS

### Baseline Characteristics

We conducted our study between April–October 2017. Of the 260 patients who were eligible for inclusion, 12 declined to participate. Consequently a total of 248 patients were included and randomized. The mean age was 61 ± 19 years, and 115 (46.4%) patients were male. Demographic and clinical characteristics at baseline were similar between the two groups (Table 2). There was no difference in time of ED arrival. There were more patients triaged as level 3 and patients with no medical conditions in the POCT group. Fever was the most commonly observed chief complaint in the study population. Disposition rate was similar between the two groups.

### Primary outcomes

Median TOD in the POCT group and CLT group were 106.50 minutes (IQR 78.25–140) and 204.50 minutes (IQR 165–244), respectively (*p* <0.001) (Table 3). Median ED LOS was also significantly shorter in the POCT group (240 minutes (IQR 161.25–410) vs 395.50 minutes (IQR 278.50–641.25); *p* <0.001). Arrival to time of first physician assessment, time for the physician assessment to draw blood, and result reporting to decision-making time were not significantly different between the two groups. However, time from first physician assessment to decision-making was significantly shorter in the POCT group (70 minutes (IQR 53.50–115.50) vs 169.50 minutes (IQR 141–208); p <0.001), as well as the overall time from decision-making to ED disposition time (117.50 minutes (IQR 30.50–298.75) vs 185.50 minutes (IQR 100.75–389.25); *p* =0.001). Additionally, the lab turnaround time of the POCT group was shorter ((5 minutes (IQR 4–6) vs 87.5 minutes (IQR 70–103).

### Secondary outcome

#### Satisfaction

The POCT system was rated as excellent and had a higher satisfaction score from physicians (84.7% vs 16.1%, *p* <0.001), nurses (68.5% vs 50.0%, *p* = 0.001) and patients (71.8% vs 46.8%, *p* <0.001) (Table 4).

## DISCUSSION

In this randomized control trial, the application of POCT resulted in a reduction in TOD and ED LOS. To our knowledge, this was the first study comparing a newly-developed POCT device to the CLT in a major university hospital in Thailand. Our results were concordant to the initial hypothesis that POCT cartridges consisting of basic metabolic panels would be sufficient for the physicians to make earlier treatment decisions. Moreover, there was still a 155.5-minute decrease in median LOS compared to the CLT group, even though 98 of 124 patients in the POCT group also required other central lab tests. This might have been because those other tests were mainly complete blood count, whose results were usually delivered earlier than electrolytes. However, our findings are in contrast with the studies by Kendall et al^16^ and Parvin et al^17^ in which POCT did not have a significant impact on ED LOS. Those authors postulated that the lack of significant impact was due to multiple factors such as unavailability of medical personnel and hospital access block, which did not occur in our study. Moreover, this contrasting result might have been due to the fact that there were more patients with no comorbidities in the POCT arm in our study, making it easier for the physicians to make their decisions and thereby facilitating faster ED disposition.

Additionally, turnaround time was significantly reduced from 87.50 minutes in the CLT to five minutes in the POCT group. This finding was similar to a previous study by Nørgaard et al,^13^ which demonstrated a decreased turnaround time by almost 45 minutes with the use of POCT. Reduced turnaround time may allow patients to receive earlier treatment, especially for emergency patients who required immediate management. Furthermore, since POCT can be performed and interpreted bedside, it helps to minimize transport distance and time to the central lab. It also helps to reduce documentation and delay and minimize the risk of wrong designation. From our results, there was an additional transfer time of 21 minutes from the urgency room to the central lab in the CLT group. The use of POCT could eliminate that transfer time.

Of the 147 cartridges used in this study, 23 could not be analyzed by the system. Additionally, there was one case with a falsely elevated potassium value. These errors might have been caused by improper storage of the cartridge or pre-analytical errors. The cartridges must be stored at temperatures between 2°–8°Celsius (C) (35°–46°Fahrenheit (F) and should not be exposed to temperatures exceeding 30°C (86°F). The cartridges should also be used immediately after they are removed from packaging to ensure accuracy of results. Moreover, the users should be trained to avoid pre-analytical errors such as inappropriate sample collection, which can cause hemolysis and subsequently hyperkalemia. Quality system instructions must be followed strictly to ensure accuracy.

Similar to the previous study by Steindel et al,^20^ more physicians, nurses, and patients preferred the POCT system over routine lab testing. One interesting finding was that there were more physicians than nurses who rated the POCT system as excellent. This might have been because POCT could deliver fast results with only a five-minute time to analysis, therefore this might not waste their time. The nurses might prefer POCT with the same reason as physicians, however POCT could not reduce the overwhelming workload of nurses. Despite the perceived advantages of POCT, we found that personnel need to be more properly trained to use system since the number of failed cartridges was nearly 15%. Most failures occurred during the initial phase of the study. This resulted in time delays and possible additional expense that could have been avoided.

## LIMITATIONS

Because this was a single-center study, it would be difficult to generalize our results to hospitals in different settings. Second, we found that the nurses failed to use the POCT device properly in the initial phase of the study, which resulted in a high cartridge-failure rate even though they had been trained beforehand by the manufacturer’s representative. The errors were mostly blood spillage over the cartridge or too much blood inserted into the cartridge, which could have made the cartridges unanalyzable. One approach to solve this problem would be more personnel training. Nevertheless, we did not record the rate of specimen recollection or hemolyzed specimens in the CLT specimens.

Third, although POCT had higher satisfaction scores from physicians, nurses, and patients, we did not assess the validity and reliability of the satisfaction questionnaire. Lastly, our study was conducted only in patients triaged as level 3 and 4. They were the population of interest since the urgency room was crowded from these patients. In fact, the POCT system would be of most benefit in level 1 and 2 patients (eg, patients with cardiac arrest or lethal electrolyte disorders) for whom POCT could facilitate prompt diagnosis and treatment decisions. However, TOD may not change in those patients because they are usually under resuscitation and receive continuous management, and it is hard to judge which treatment decision was made based on electrolyte results.

## CONCLUSION

This study demonstrated a significant decrease in lab turnaround times, time to decision-making, and ED length of stay after the implementation of a point-of-care testing system. Physicians, nurses, and patients were more satisfied with the POCT compared to central lab turnaround times. This intervention led to better utilization of resources and more patient access, as well as faster time to decision-making and shorter lengths of stay in the ED.

## Supplementary Information



## Figures and Tables

**Table 1:** Adult triage system used in the urgency room of Siriraj Hospital, Bangkok.

Siriraj Adult Triage System*	Time to medical attention
Level 1	Immediate life-threatening conditions requiring emergent medical attention
Level 2	Emergency, requiring medical attention within 10 minutes
Level 3	Urgency, requiring medical attention within 30 minutes
Level 4	Semi-urgency, requiring medical attention within 60 minutes
Level 5	Non-urgency, requiring medical attention within 2 hours

* Patients classified as levels 1 and 2 were admitted to the emergency department while those categorized as levels 3 to 5 were transferred to the urgency room.

**Table 2:** Baseline characteristics.

Baseline Characteristics	Total	POCT, n (%)	CLT, n (%)	P-value
Patient age (in years, mean ± SD)	61 ± 19	60 ± 20	62 ± 17	0.299
Gender				0.702
Male	115 (46.40%)	56 (45.20%)	59 (47.60%)	
Arrival period				0.491
Working hour	164 (66.10%)	84 (67.70%)	80 (64.50%)	
Holiday hour	84 (33.90%)	40 (32.30%)	44 (35.50%)	
Triage				0.047
Level 3	89 (35.90%)	52(41.90%)	37 (29.80%)	
Level 4	159 (64.10%)	72 (58.10%)	87 (70.20%)	
Chief complaint				
Fatigue	31 (12.50%)	18 (14.50%)	13 (10.50%)	0.337
Diarrhea	17 (6.90%)	7 (5.60%)	10 (8.10%)	0.451
Dyspnea	19 (7.70%)	11 (8.90%)	8 (6.50%)	0.474
Alteration of consciousness	27 (10.90%)	13 (10.50%)	14 (11.30%)	0.838
Fever	49 (19.80%)	19 (15.30%)	30 (24.20%)	0.079
Dizziness	23 (9.30%)	11 (8.90%)	12 (9.70%)	0.827
Nausea and vomiting	26 (10.50%)	12 (9.70%)	14 (11.30%)	0.678
Abdominal pain	42 (16.90%)	22 (17.70%)	20 (16.10%)	0.735
Exacerbation of underlying disease	4 (1.60%)	1 (0.80%)	3 (2.40%)	0.313
Weakness	13 (5.20%)	7 (5.60%)	6 (4.80%)	0.776
Others	43 (17.30%)	25 (20.20%)	18 (14.50%)	0.240
Medical conditions				
Old CVA	16 (6.50%)	7 (5.60%)	9 (7.30%)	0.605
Dyslipidemia	42 (16.90%)	25 (20.20%)	17 (13.70%)	0.176
Diabetes mellitus	82 (33.10%)	40 (32.30%)	42 (33.90%)	0.787
Hypertension	91 (36.70%)	46 (37.10%)	45 (36.30%)	0.895
Asthma/COPD	12 (4.80%)	2 (1.60%)	10 (8.10%)	0.018
Chronic kidney disease	25 (10.10%)	11 (8.90%)	14 (11.30%)	0.527
Cirrhosis	6 (2.40%)	1 (0.80%)	5 (4.00%)	0.098
Malignancy	39 (15.80%)	21 (17.10%)	18 (14.50%)	0.582
Cardiovascular disease	50 (20.20%)	25 (20.20%)	25 (20.20%)	1
No medical conditions	45 (18.20%)	29 (23.60%)	16 (12.90%)	0.03
Disposition				0.496
Discharge	172 (69.4%)	83 (66.90%)	89 (71.80%)	
Transfer to the ED	19 (7.7%)	9 (7.30%)	10 (8.10%)	
Refer to other hospital	10 (4.0%)	4 (3.20%)	6 (4.80%)	
Admit to ward	47 (19.0%)	28 (22.60%)	19 (15.30%)	

*POCT*, point-of-care testing; *CLT*, central laboratory testing; *CVA*, cerebrovascular accident; *COPD*, chronic obstructive pulmonary disease; *ED*, emergency department.

**Table 3:** Time difference between point-of-care testing and central lab testing.

	Time in minutes, median (IQR)		
	POCT	CLT	P-value
Primary outcomes			
Arrival to time of decision-making	106.50 (78.25–140.00)	204.50 (165.00–244.00)	<0.001
ED length of stay	240 (161.25–410.00)	395.50 (278.50–641.25)	<0.001
Time intervals			
Arrival to physician assessment time	25.00 (15.00–42.25)	25.00 (15.00–39.75)	0.571
Physician assessment to blood draw time	36.50 (23.00–51.00)	32.50 (25.00–50.00)	0.685
Physician assessment to decision-making time	70.00 (53.50–115.50)	169.50 (141.00–208.00)	<0.001
Result reporting to decision-making time	10.00 (5.00–49.75)	15.00 (10.00–20.00)	0.139
Decision-making to ED disposition time	117.50 (30.50–298.75)	185.50 (100.75–389.25)	0.001
Laboratory turnaround time			
POCT group	5.00 (4.00–6.00)	-	N/A
CLT group	-	87.50 (70.00–103.00)	N/A
Blood draw to complete laboratory time*	72.00 (54.50–90.00)	87.50 (70.00–103.00)	<0.001

* Defined as the period between time of blood draw to the time all the results were reported.

*IQR*, interquartile range; *POCT*, point-of-care testing; *CLT*, central laboratory testing.

**Table 4:** Satisfaction scale.

Satisfaction scale	POCT, n(%)	CLT, n(%)	p-value
Physician satisfaction			<0.001
Good	18 (14.50%)	63 (50.80%)	
Excellent	105 (84.70%)	20 (16.10%)	
Nurse satisfaction			0.001
Good	38 (30.60%)	51 (41.10%)	
Excellent	85 (68.50%)	62 (50.00%)	
Patient satisfaction			<0.001
Good	33 (26.60%)	54 (43.50%)	
Excellent	89 (71.80%)	58 (46.80%)	

*POCT*, point-of-care testing; *CLT*, central laboratory time.
