# Supplementary material for: Role of Point-of-Care Testing in Reducing Time to Treatment Decision-Making in Urgency Patients: A Randomized Controlled Trial
Source: West J Emerg Med. 2020 Feb 25;21(2):404–10. doi: 10.5811/westjem.2019.10.43655 (PMC7081845; doi:10.5811/westjem.2019.10.43655)
Supplement: Supplementary file 1 [file wjem-21-404-s001.docx]

**Supplementary Appendix**

**Table S1.** Satisfaction scale.

| Grade | Scale | Definition |
| --- | --- | --- |
| 5 | excellent | Very easy to use, very fast results, minimize waiting time |
| 4 | good | Easy to use, faster results, minimize waiting time |
| 3 | satisfactory | Easy to use but not different waiting time or  Difficult to use but minimize waiting time |
| 2 | poor | Difficult to use, not different waiting time |
| 1 | very poor | Very difficult to use, inconvenient, not different waiting time |
